# Supplementary material for: HIF-1α Causes LCMT1/PP2A Deficiency and Mediates Tau Hyperphosphorylation and Cognitive Dysfunction during Chronic Hypoxia
Source: Int J Mol Sci. 2022 Dec 17;23(24):16140. doi: 10.3390/ijms232416140 (PMC9783654; doi:10.3390/ijms232416140)
Supplement: Supplementary file 1 [file ijms-23-16140-s001.zip › ijms-2032562-supplementary.pdf]

Supplementary 1. Chronic hypoxia promotes the increase of HIF-1α expression in the nuclear.

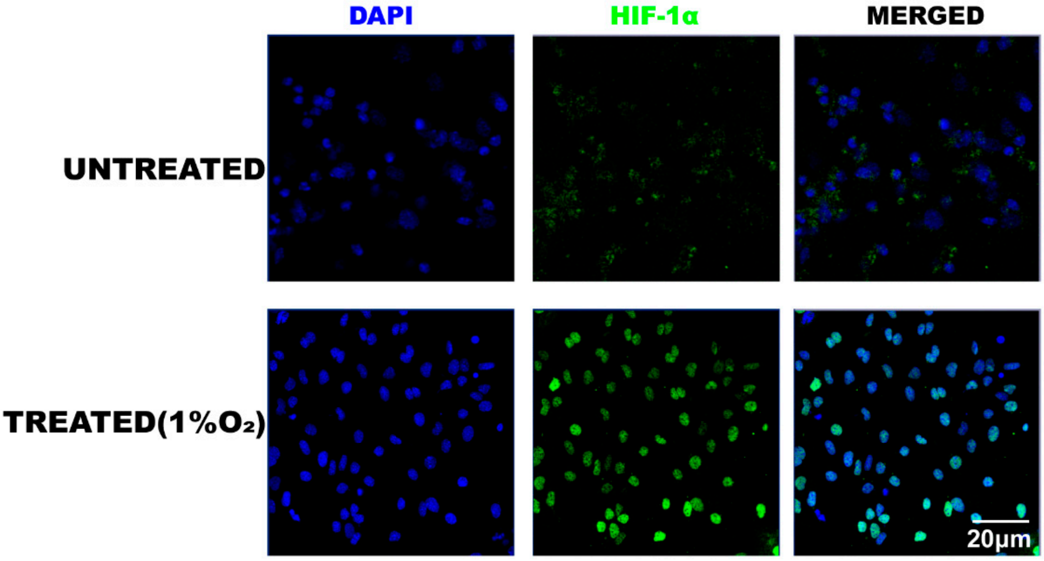

Figure S1. Immunofluorescence assay showed a marked nuclear translocation of HIF-1α. (scale bars=20μm)

Supplementary 2. Hypoxia upregulates HIF-1α and leads to tau hyperphosphorylation in C6/tau cells.

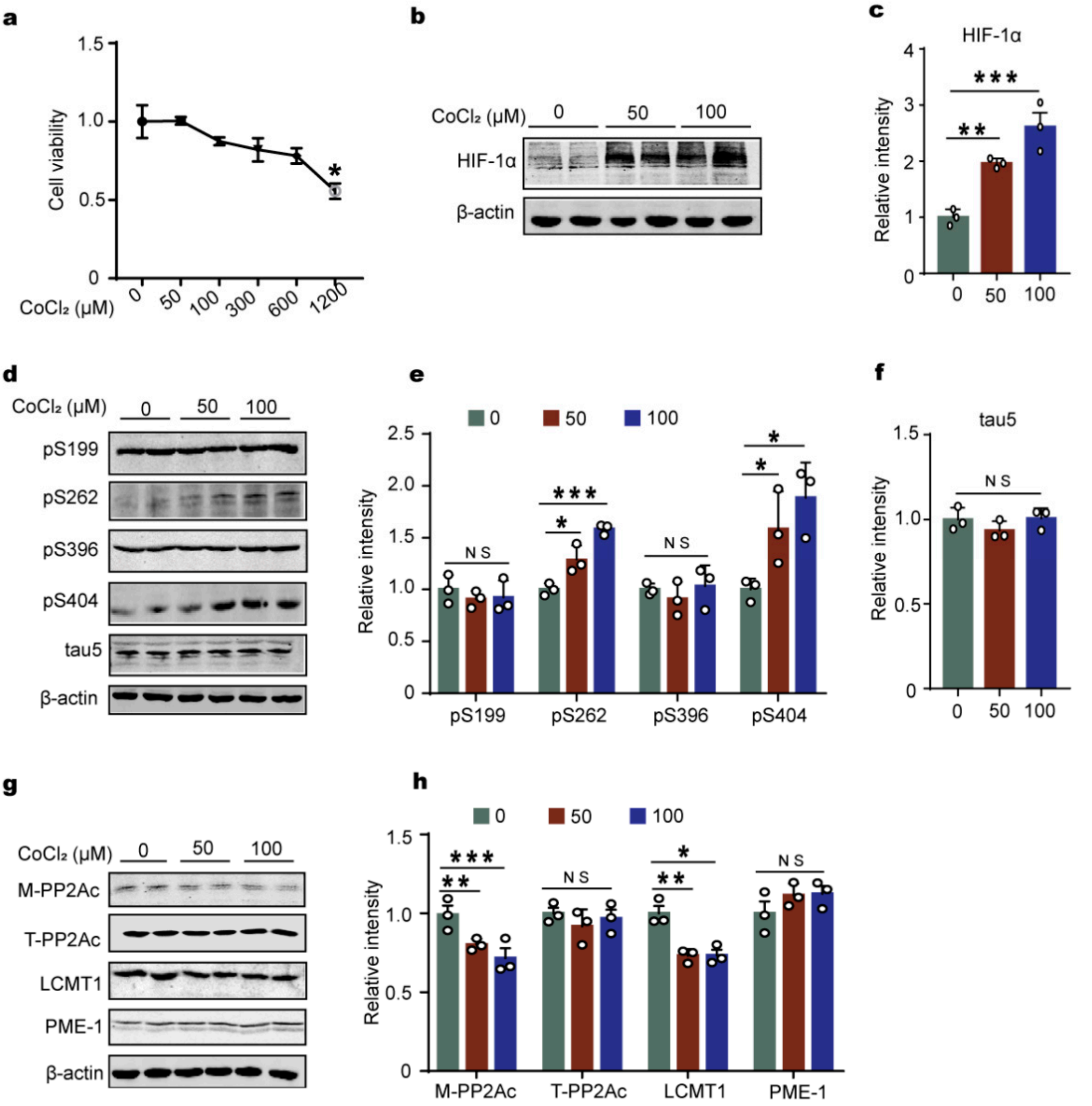

Figure S2. Chemical hypoxia upregulates HIF-1α, resulting in LCMT1/PP2A deficiency and tau hyperphosphorylation in C6/tau cells. (a) CCK8 assay in C6/tau cells treated with increasing doses of $CoCl_2$ (0, 50, 100, 300, 600, 1200 μmol/L) for 8h. (b) Western blots for HIF-1α in C6/tau cells treated with $CoCl_2$ (0, 50, 100 μmol/L) for 8h. (c) Quantification of the relative protein expression levels HIF-1α after normalization to the β-actin signal. (d) Western blots for tau phosphorylation levels at different sites including pS199, pS262, pS394, pS404, and tau5 in C6/tau cells treated with $CoCl_2$ (0, 50, 100μmol/L) for 8h. (e, f) Quantification of the relative protein expression levels (pS199, pS262, pS396, pS404) and tau5 normalization to the β-actin signal. (g, h) Western blots and quantitative analysis of T-PP2Ac, M-PP2Ac, LCMT1, and PME-1 in C6/tau cells treated with $CoCl_2$ (0, 50, 100 μmol/L) for 8 h. Data represent mean ± SD, n = 3, *$p < 0.05$, **$p < 0.01$, ***$p < 0.001$ vs control.

Supplementary 3. The plasmid (6315) is the highest efficiency inhibitor of HIF-1α among three siHIF-1α plasmids.

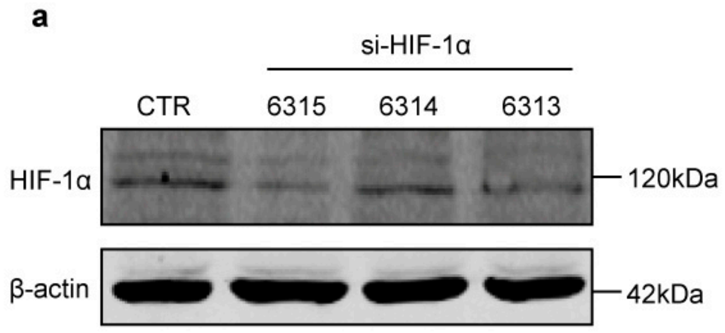

Figure S3. The plasmid (6315) is the highest efficiency inhibitor of HIF-1α in the **three siHIF-1α plasmids.** (a) Western blots for HIF-1α in C6 cells transfected with the three si-HIF-1α plasmids.
